# Comparison of Ultrasonography, Contrast Radiographic Tenography, Cone-Beam Computed Tomographic Tenography, and Tenoscopy for Lesion Detection Within the Digital Flexor Tendon Sheath of Horses—A Prospective Clinical Trial

**DOI:** 10.3390/vetsci13030268

**Published:** 2026-03-13

**Authors:** Cassandra B. Sapper, Christoph Koch, Daniela Schweizer, Laura Cunha Silva, Frederik E. Pauwels, Micael D. Klopfenstein, Mathieu de Preux, Elke Van der Vekens

**Affiliations:** 1 Division of Equine Surgery, Swiss Institute of Equine Medicine, Department of Clinical Veterinary Science, Vetsuisse Faculty, University of Bern, 3012 Bern, Switzerland; cassandra.sapper@hotmail.com (C.B.S.); christoph.koch@unibe.ch (C.K.); micael.klopfenstein@unibe.ch (M.D.K.); mathieu.depreux@unibe.ch (M.d.P.); 2 Division of Clinical Radiology, Department of Clinical Veterinary Science, Vetsuisse Faculty, University of Bern, 3012 Bern, Switzerland; daniela.schweizer@unibe.ch; 3 Veterinary Public Health Institute, Department of Clinical Research and Veterinary Public Health, Vetsuisse Faculty, University of Bern, 3012 Bern, Switzerland; laura.dasilva@unibe.ch; 4 Plexus Veterinary Imaging, Palmerston North 4410, New Zealand; fred@plexusveterinaryimaging.com

**Keywords:** horse, deep digital flexor tendon, superficial digital flexor tendon, manica flexoria

## Abstract

Lesions of the tendons and manica flexoria (MF) within the digital flexor tendon sheath (DFTS) are a common cause for lameness in horses. This prospective study compared and quantified the agreement and disagreement of positive contrast computed tomographic tenography (CTT), positive contrast radiographic tenography (RXT), ultrasonography (US) and tenoscopy for diagnosing naturally occurring lesions within the DFTS, without application of a gold standard. Lesions affecting the deep (DDFT) and/or superficial digital flexor tendon (SDFT), and/or the MF and/or constriction of the palmar/plantar annular ligament (PAL) were evaluated in eighteen horses with distention of the DFTS. For DDFT lesions, comparing CTT with tenoscopy, US, and the combined results of US and RXT (US+RXT) attained the highest agreements, with 83% matching results (κ: 0.65). For SDFT lesions, CTT and tenoscopy showed the highest agreement with 94% matching results (κ: 0.89), followed by tenoscopy with US+RXT (78%; κ: 0.56). The highest agreement for MF-tear detection was found comparing CTT with tenoscopy (83%; κ: 0.67), followed by CTT with RXT (78%; κ: 0.56). None of the modalities agreed on positive diagnoses of PAL constriction. CTT achieved the highest agreement with tenoscopy and US for the diagnosis of lesions within the DDFT and is, therefore, considered the most useful modality for preoperative evaluation.

## Simple Summary

Lesions of the superficial (SDFT) or deep (DDFT) digital flexor tendons, manica flexoria (MF) or palmar/plantar annular ligament (PAL) within or adjacent to the digital flexor tendon sheath (DFTS) are common causes of lameness in horses. A comprehensive diagnostic imaging workup is critical for accurately defining the underlying cause and ensuring successful case management. This prospective study compared and quantified the agreement between different imaging modalities and tenoscopy. The imaging included radiography (RXT) and computed tomography (CTT), with contrast medium injected into the DFTS to visualize the tendons and MF, and ultrasonography. Eighteen horses with naturally occurring DFTS distention were evaluated. For DDFT, SDFT and MF lesions, the highest agreement was found between CTT and tenoscopy. Agreement between imaging modalities for the diagnosis of PAL constriction (PALco) proved to be poor, as none of the modalities agreed on affected cases. Therefore, CTT is considered the best modality to diagnose the lesion causing distention of the DFTS when the SDFT, DDFT or MF are affected, showing lesions unreported both on ultrasonography and tenoscopy. Combining RXT and ultrasonography increased the agreement with CTT compared to either modality alone and should be considered when CTT is not available pre-surgery.

## 1. Introduction

The equine digital flexor tendon sheath (DFTS) extends from the distal half of the metacarpus/-tarsus to the level of the distal interphalangeal joint on the palmar/plantar aspect of the extremity. It contains the superficial digital flexor tendon (SDFT), the deep digital flexor tendon (DDFT), and their manicae, vinculae, and mesotenons. The proximal manica flexoria (MF) of the SDFT surrounds the DDFT proximal to the proximal sesamoid bones. At the level of the fetlock, the flexor tendons are held in place by the palmar/plantar annular ligament (PAL), which inserts on the abaxial margins of the proximal sesamoid bones. In the pastern, the palmar/plantar wall of the DFTS is reinforced by the proximal digital annular ligament. The DFTS forms several recesses, including palmaro-/plantaro- and dorsoproximal; collateral; and palmaro-/plantaro- and dorsodistal recesses [1].

Pathologies of structures contained within the DFTS are common and can lead to effusion of the DFTS and lameness of varying degrees [2]. The most common causes of non-septic tenosynovitis are marginal tears of the DDFT and the MF [2,3]. Tears of the DDFT predominantly occur in forelimbs [4,5], specifically on the lateral border at the level of the MF [6,7]. They are commonly found in thoroughbreds, warmbloods, and draft horses [7,8]. Tears of the MF predominantly occur in hindlimbs and are frequently seen in ponies and cobs [4,5,8,9,10]. Contradictory information regarding the location of MF tears exists. Whereas one group of investigators observed that MF tears are most frequent on the medial aspect [2,5,9,10,11], others report that MF tears more frequently occur on the lateral aspect [12,13] or that they occur equally frequently medially and laterally [7].

Other non-infectious pathologies involving the DFTS include tendonitis of the DDFT or SDFT [6,7,14,15] and desmitis or constriction of the PAL [16]. Injuries of the SDFT in the pastern region are reportedly more common in forelimbs and are frequently seen in eventers, steeplechasers, and trotters [17,18]. Again, the lateral aspect of the SDFT seems to be more prone to injury [2]. Desmitis of the PAL can be either primary or secondary, with primary desmitis being caused by direct or indirect trauma. Secondary desmitis of the PAL can be caused by tendonitis of the DDFT or SDFT or by tenosynovitis of the DFTS. Both forms of desmitis can lead to thickening of the PAL and subsequently to constriction of the digital flexor tendons [19].

Horses with DFTS pathology can be managed either conservatively or surgically. The decision for one or the other is complex. However, knowing what structures are involved and the lesion severity can be important factors in this decision process, as those factors influence the prognosis. When evaluating the return to previous levels of work among lesions managed by tenoscopic debridement, a less favorable prognosis was reported in horses with marginal tears of the DDFT (36.6–42%) [2,4,6] compared to horses with tears of the MF (55.5–79%) or horses that had a PAL desmitis in addition to adhesions or synovial masses (72%) [9,19]. Therefore, a comprehensive preoperative diagnosis is important to inform owners about prognosis [9,20].

Different modalities have been used to evaluate pathologies in the DFTS with reported sensitivities and specificities, which vary between studies and depend on the affected structure, as well as the nature of the lesion. Studies calculating the sensitivity (Se) and specificity (Sp) require a gold-standard diagnostic modality for comparison, for which tenoscopy is usually used. Ultrasonography (US) is the most widely used modality to assess pathologies in the DFTS. However, it reportedly provides an underestimation of the extent of tendinous damage [7,21], specifically for tears of the MF and the DDFT [2,4,9,10,22]. In this context, it needs to be considered that the recently described dynamic ultrasound technique for the assessment of the DFTS was not applied in any of these studies, which may improve recognition of MF tears [12,22,23]. In addition, US can be used for the diagnosis of PAL desmitis [16,24].

Positive contrast radiographic tenography (RXT) has gained increasing importance in complementing US for the diagnostic workup of DFTS pathologies in horses [3,8]. Although RXT has a low Se for the diagnosis of DDFT tears, it has a high Se in diagnosing MF tears, albeit a lower Sp than US for both these structures [3,4,8]. Furthermore, RXT of the DFTS is restricted to lateromedial projections, and therefore, it is impossible to determine the laterality of a lesion without additional imaging. Nonetheless, the combination of US and RXT has been shown to increase the likelihood of lesion identification, particularly for MF tears [4].

Two recent studies describe the use of positive contrast computed tomographic tenography (CTT) of the DFTS in cadaveric specimens obtained from horses without DFTS pathology [25,26]. Both experimental studies confirm that with CTT, the soft tissue-attenuating structures contained within the DFTS are outlined by hyperattenuating contrast medium. Therefore, CTT allows for detailed anatomical visualization and assessment of the contour, shape and size of these structures [25,26]. In another experimental study performed on cadaveric specimens, CTT allowed for the diagnosis of surgically induced SDFT and MF lesions with a high Se and Sp and DDFT lesions with a high Sp [27]. In addition, examination of the distal limb using CTT is feasible in standing sedated horses and can contribute valuable information regarding lesions within the DFTS [28].

Tenoscopy is still considered the most reliable technique for the diagnosis of MF and marginal DDFT and SDFT tears within the DFTS [2,29]. With a systematic approach, tenoscopy allows for a complete examination of the DFTS and its contents [15]. Although tenoscopy is often set as the gold standard modality for the diagnosis of pathologies within the DFTS [3,4,8], it is restricted to evaluating the surfaces of the anatomical structures and not their internal integrity.

The objective of the present study is to compare and quantify the agreement between US, RXT, CTT, and diagnostic tenoscopy for the diagnosis of pathologies of the DDFT, SDFT, and MF within the DFTS and PAL constriction (PALco) without relying on tenoscopy as a gold standard. We hypothesized that CTT would detect more defects than all other tested imaging modalities for all lesions reaching the surface of the anatomical structures contained within the DFTS and would show the highest agreement with tenoscopy.

## 2. Materials and Methods

### 2.1. Study Population and Inclusion Criteria

Horses presented to the Swiss Institute of Equine Medicine at the University of Bern, Switzerland, between August 2016 and January 2023 for evaluation of synovial distention of the DFTS, with or without associated lameness, were considered for inclusion in this prospective study. To be included, the owners needed to agree that their horses underwent all the following imaging modalities: US, radiography, RXT, and standing CTT, as well as DFTS tenoscopy. The full medical records of these horses were collected. The recorded data comprised the horse’s age, breed, weight, gender, clinical findings, and findings from the above-mentioned modalities. The recorded clinical findings included the degree of lameness according to AAEP lameness grading [30], affected limb, grade of DFTS distention, duration of lameness, response to flexion test, and response to intrathecal anesthesia of the DFTS.

### 2.2. Diagnostic Imaging Techniques

All horses underwent the diagnostic modalities in the same order—US, radiography, RXT, and CTT—either on the same day or US one day prior to the other imaging modalities. In all modalities, a full evaluation of the obtained images was performed, but only information regarding tendinopathy of the SDFT and DDFT, pathology of the MF, and PALco was further evaluated in this study. For all modalities, all abnormal findings, including tears, border irregularities, fibrillation, and thickening of the SDFT, DDFT and MF were recorded as a lesion for that structure in that horse. This was independent of the number of modalities in which the lesion was observed. Further specifications about lesion detection are described for each modality.

On every horse, US was performed, and all radiographs and CT studies were assessed by one of three radiologists (one board-certified and two board-eligible radiologists of the European College of Veterinary Diagnostic Imaging), with the same radiologist assessing all imaging studies for the same horse. The observers were neither blinded to the findings of the clinical examination nor to the previous imaging findings. All images were reviewed using the same imaging software (DeepUnity Diagnost, 1.2.0.1, Dedalus Healthcare, Bonn, Germany) on radiology workstations equipped with screens designated for medical image analysis and reported in the clinical information system.

All horses were sedated using a standardized protocol, combining detomidine hydrochloride (0.01 mg/kg IV) (Equisedan, Graeub Veterinary Products, Bern, Switzerland) with butorphanol (0.01 mg/kg IV) (Morphasol-10, Graeub Veterinary Products, Bern, Switzerland). Additional top-ups of the same combination were given if needed.

#### 2.2.1. Ultrasonography

Ultrasonographic examination of the DFTS, and the structures contained within the DFTS, was performed with one of three available US machines (GE Healthcare LOCIQe or GE Healthcare LOGIQ S8, GE Medical Systems AG, Glattbrugg, Switzerland; or Siemens ACUSON Sequoia, Siemens AG, Forchheim, Germany) and using multifrequency linear transducers (5–13 MHz, 5–8 MHz, 2–8 MHz, or 9–12 MHz). The palmar/plantar metacarpal/-tarsal, fetlock, and pastern regions were clipped and cleaned with warm water and soap, and ultrasound gel was applied. The affected limb was first examined while weight-bearing, followed by static and dynamic non-weight-bearing examinations. If considered beneficial, a stand-off pad was used. During the weight-bearing and static non-weight-bearing examination, the SDFT, DDFT, MF, and PAL were evaluated, , while the presence of the gliding motion of the SDFT relative to the PAL and the DDFT was assessed during the dynamic examination. The complete US examination also included all structures adjacent to the DFTS, and these findings were recorded (Supplementary Table S1). However, these structures were not included in the analysis comparing the different imaging modalities.

As previously described [12], SDFT and DDFT lesions were diagnosed when the tendons showed an altered transverse cross-sectional appearance, when areas of reduced/increased echogenicity and disruption of architecture were present, or when focal increased vascularization was observed during Doppler examination. Lesions of the MF were suspected in the presence of a loose and/or asymmetric appearance, heterogeneous echogenicity, and irregular margin definition. The PAL was considered “thickened” if it had an abnormal appearance with diffuse or focal decrease in echogenicity and loss of fiber pattern, which was associated with a palmaro-/plantarodorsal thickness exceeding 1.6 mm in the midline or the measurement near the insertion on the proximal sesamoid bones exceeding 5 mm, consistent with previous studies [16,19]. Attention was paid to distinguishing the PAL from subcutaneous tissues before measuring its thickness to avoid the common error of measuring both [21]. Constriction was diagnosed when the gliding motion of the flexor tendons appeared to be restricted during the dynamic examination.

#### 2.2.2. Radiography

For each horse, four standard radiographic projections of the fetlock region were acquired: lateromedial (LM), dorsopalmar/-plantar (DPa/DPl), dorsal 45°lateral–palmaro-/plantaromedial oblique (DL-PaMO/DL-PlMO), and dorsal 45° medial–palmaro/plantarolateral oblique (DM-PaLO/DM-PlLO). These four native projections were performed with a ceiling-mounted X-ray generator (Siemens Polydoros SX 65/80, Siemens AG, Forchheim, Germany) and a computed radiography system (Fujifilm, FCR Profect CS Plus, digitalXray AG, Niederscherli, Switzerland). For the subsequent RXT and CTT, the sedated horse was placed in stocks. After aseptic preparation, 40 mL of 100 mg/mL diluted non-ionic iodinated contrast medium (300 mg/mL Iohexol (Accupaque 300, GE Healthcare, GE Medical Systems AG, Glattbrugg, Switzerland), in a 1:2 dilution with sterile 0.9% sodium chloride solution, was injected into the distal palmar/plantar recess of the DFTS using a 20-gauge 38 mm needle. The distal limb was flexed and extended passively to distribute the contrast medium. A portable X-ray tube (Gierth TR 90/30, Gierth X-Ray International GmbH, Riesa, Germany) was used in combination with a direct digital radiography system (Fujifilm FDR D-EVO plus C24i, digitalXray AG, Niederscherli, Switzerland) to perform a weight-bearing LM projection centered on the fetlock.

**Figure 1:**
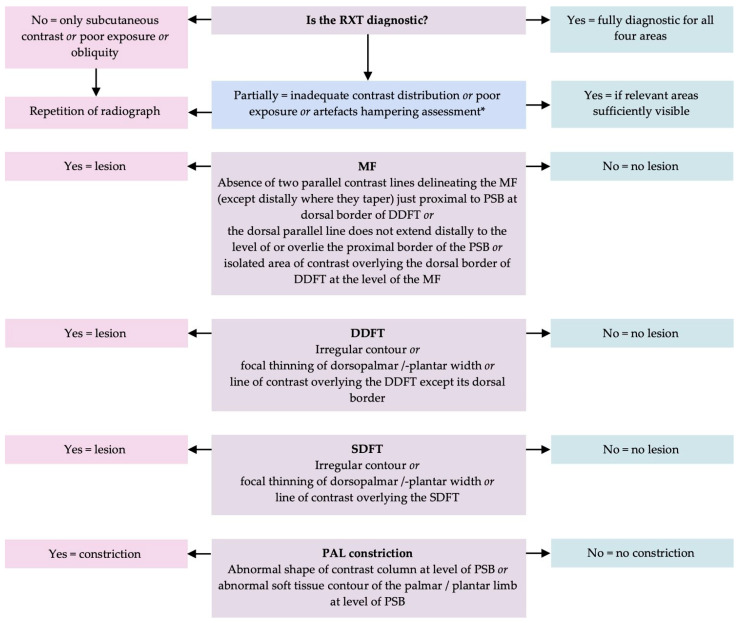
Flowchart for the diagnosis of a lesion of the manica flexoria (MF), deep digital flexor tendon (DDFT) and superficial digital flexor tendon (SDFT) within the digital flexor tendon sheath (DFTS) and palmar/plantar annular ligament (PAL) in positive contrast radiographic tenography (RXT), adapted after Kent et. al., 2020 [8] . * RXT with subcutaneous leakage was not repeated, but this was considered a limitation of this modality. Further abbreviations: proximal sesamoid bone (PSB).

Figure 1 shows the diagnostic algorithm and criteria used to assess the RXT, adapted from Kent and collaborators [8], and to diagnose lesions of the DDFT, MF, and PALco. For the evaluation of the SDFT, in particular for diagnosing lesions of the SDFT, the observers applied criteria according to those described for DDFT lesions.

#### 2.2.3. Computed Tomography

**Figure 2:**
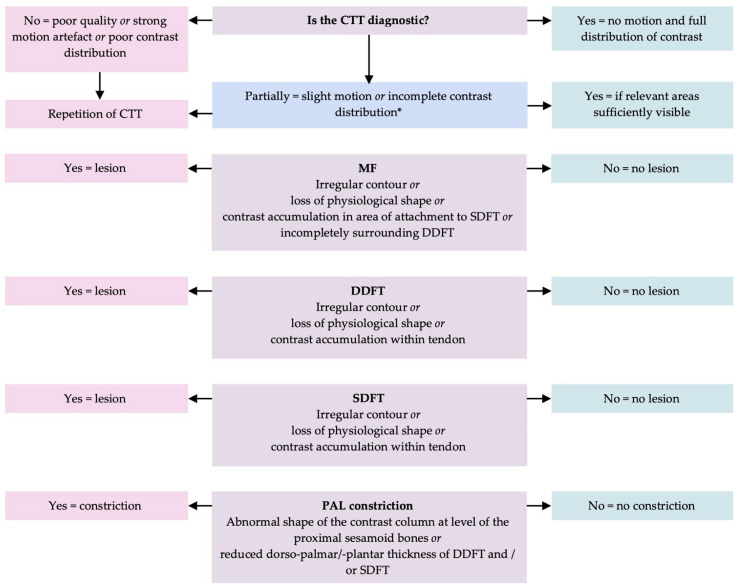
Flowchart for the diagnosis of a lesion of the manica flexoria (MF), deep digital flexor tendon (DDFT) and superficial digital flexor tendon (SDFT) within the digital flexor tendon sheath (DFTS) and palmar/plantar annular ligament (PAL) in positive contrast computed tenography. * If mild motion was present on repeated scans, despite adequate sedation, and they were considered diagnostic by the radiologist, no further repetitions were acquired in accordance with the ALARA principle of radioprotection.

All horses underwent standing cone-beam computed tomography (CBCT) of the DFTS region using one of two available CBCT units (O-arm; O-arm O2, Medtronic inc., Medtronic AG, Münchenbuchsee, Switzerland). In each horse, the CTT study was performed immediately following RXT, under the same sedation and without additional contrast medium being injected. Isovolumetric images were reconstructed using a bone algorithm in a slice thickness of 0.833 mm and analyzed using multiplanar reconstructions. Descriptions of CTT imaging of clinically healthy fore- and hindlimbs of horses [25,26] were used as a reference for the assessment of the CTTs. Figure 2 shows the diagnostic algorithm and criteria used for the assessment of the CTTs to diagnose lesions of the MF, DDFT, SDFT, and PALco.

### 2.3. Tenoscopy

Diagnostic tenoscopic exploration was performed under general anesthesia using a previously described standard protocol [15,31]. All but three horses were operated on one to four days after the diagnostic imaging was performed. In three horses, a conservative treatment with intrasynovial triamcinolone injection and rest was attempted after the diagnostic imaging and diagnosis, but it did not lead to improvement of lameness. These horses underwent surgery one (horse 3), four (horse 1), and six (horse 12) months later. All surgeries were performed by one of five staff surgeons employed at the ISME Equine Clinic Bern during the study period, all of whom are board-certified surgeons with the American and/or European College of Veterinary Surgeons. The surgeons were not blinded to the findings of the clinical examination or the imaging findings. The surgeon reported the results of the tenoscopy in the clinical information system. Tenoscopy was performed with the horse positioned in left or right lateral recumbency based on the findings of the preoperative diagnostic imaging.

The standard approach through the collateral and dorsoproximal recess of the affected side (lateral or medial) was used, with additional portals created if needed. The initial standard scope portal was in the outpouching of the DFTS between the PAL and proximal digital annular ligament (the collateral recess), slightly palmar/plantar to the neurovascular bundle, and just distal to the PAL. A 4 mm 30° forward oblique arthroscope (Hopkins II, Karl Storz AG, Anklin AG, Binningen, Switzerland) was introduced to explore the DFTS. Lesions of the SDFT, DDFT, or MF, as well as additional lesions, were recorded. Lesions of the flexor tendons or partial lesions of the MF were debrided using an arthroscopic shaver (Shaver Power System II, Arthrex, Arthrex Swiss AG, Belp, Switzerland). Constriction of the PAL was diagnosed when the operating surgeon encountered difficulties in advancing the endoscope through the fetlock canal. In this case, or if the previous diagnostic imaging was suggestive of PALco, a PAL desmotomy was performed under endoscopic control with a hook knife as described [15]. In cases with total MF tears, the MF was resected following PAL desmotomy and after creating additional portals as needed [15]. All portals were closed in a routine manner.

### 2.4. Image and Report Review

To address a possible observer bias, one board-certified radiologist (EVdV) and one board-certified surgeon (CK) reviewed the stored images available for both tenographies and tenoscopies. When, during this review, previously unreported lesions were observed in RXT, CTT or tenoscopy, the results were adjusted. Thereafter, the cases where imaging and surgical findings differed were discussed, and a consensus results table was created, serving as a base for further statistical evaluations. As only still images or short videos showing the observed lesions were available for multiple ultrasonographic examinations, the results for this modality were not re-evaluated.

### 2.5. Statistical Analysis

For each assessed anatomical structure (DDFT, SDFT, MF, and PAL) and each of the four modalities (US, RXT, CTT, tenoscopy), binary outcomes reflecting whether a lesion was observed or not were recorded by the first author based on the consensus results table, created as described in Section 2.4. Furthermore, the differentiation between lateral and medial lesion location for the DDFT, SDFT, and MF established by US, CTT, and tenoscopy was recorded for descriptive statistics. In addition, the proximodistal localization (metacarpus/-tarsus versus pastern level) of tendon lesions was recorded but not included in the statistical analysis. All data was collected in Microsoft Excel tables (Excel 2016, Windows 11 Enterprise, Microsoft Corporation, Redmond, Washington, DC, USA), and statistical analyses were run using the NCSS 2024 Software (NCSS, LLC, Kaysville, UT, USA, ncss.com/software/ncss). Agreement statistics for all modalities and each lesion localization were calculated using McNemar tests, with a significance level set to *p* < 0.05, and Cohen’s Kappa statistics, including their 95% confidence intervals. The outcomes of US and RXT were combined for additional agreement statistics in comparison to CTT and tenoscopy. The agreement classification was adapted from Landis and Koch [32]. Kappa values (κ) less than 0.40 were defined as poor agreement, values between 0.41 and 0.60 as moderate agreement, values between 0.61 and 0.80 as good agreement, and values greater than 0.81 as very good to excellent agreement between the two modalities [33]. Furthermore, the “proportions matching” and “proportions non-matching” were calculated for each modality comparison and lesion localization. The proportions matching demonstrate to what extent the compared modalities agreed on the diagnosis, meaning the presence and absence of a lesion. The proportions non-matching show in which percentage of cases the compared modalities disagreed; i.e., in one modality, a lesion was diagnosed, and in the other, no lesion was seen. A sample size calculation was performed using the PASS Software ( Power Analysis and Sample Size Software (2024). NCSS, LLC. Kaysville, UT, USA, ncss.com/software/pass). A sample size of 18 subjects results in a two-sided 95% confidence interval with a width of 0.185 if the value of κ is 0.400 and the standard deviation, SD (κ), is 0.200 [34].

## 3. Results

Eighteen horses, ten mares and eight geldings, met the inclusion criteria. Ages ranged from 5 to 18 years (median age: 11.5 years). Nine horses (50%) were warmbloods, three were Franches-Montagnes horses (16.7%), and six horses (33.3%) were of other breeds (one Standardbred, one Criollo, two Icelandic horses, one Anglo-Arabian, and one Camargue horse). The median weight was 578 kg (range 319–711). Nine fore (50%) and nine hind (50%) limbs were affected, with right limbs more commonly affected than left limbs (right fore: *n* = 7, right hind: *n* = 4, left fore: *n* = 2, and left hind: *n* = 5). Lameness was chronic in all but two horses, which presented with lameness of less than two weeks’ duration. Nine horses were lame for two months or longer. In the remaining seven horses, the duration of lameness ranged between two weeks and two months. Lameness severity scores ranged from 0/5 to 3/5, with twelve horses (66.7%) graded with a 3/5 lameness score, four horses (16.7%) with a grade 2/5, and one horse each (5.6%) with a grade 1/5 or 0/5. Swelling of the DFTS was present in all 18 horses: ten horses (55.6%) showed severe, five (27.8%) moderate, and three (16.7%) mild distention of the DFTS. The flexion test of the affected limb was positive in all horses. Intrathecal anesthesia was applied to twelve horses (66.7%) and was positive in all of them. No intrathecal anesthesia was performed in three horses (16.7%), and in the remaining three horses (16.7%), no information regarding diagnostic anesthesia was available. All horses except three were examined using the same ultrasound system (GE Healthcare LOGIQ S8, GE Medical Systems AG, Glattbrugg, Switzerland). In horses 1 and 3, the GE LOCIQe (GE Healthcare LOCIQe, GE Medical Systems AG, Glattbrugg, Switzerland) unit was used, and in horse 2, a Siemens ACUSON Sequoi (Siemens AG, Forchheim, Germany) ultrasound system was used. The first 15 horses underwent CTT using the O-arm (Medtronic inc., Medtronic AG, Münchenbuchsee, Switzerland), which was replaced by the O-arm O2 (Medtronic inc., Medtronic AG, Münchenbuchsee, Switzerland) for the last three horses (Horses 16–18).

All horses underwent diagnostic tenoscopy of the affected digital sheath. The duration of general anesthesia ranged from 105 to 260 min (median 155 min). In one horse, tenoscopy was stopped due to substantial extravasation, which impeded adequate exploration. Therefore, diagnostic and surgical tenoscopy were repeated one month later. In two horses, tenoscopy was converted to an open approach after complete diagnostic exploration of the digital sheath. In one of these two horses, the surgeon elected to enlarge the scope portal to facilitate adhesion breakdown between a large lesion of the SDFT and the sheath wall, at the level close to the scope portal and the base of the proximal sesamoid bone. For the other horse, it was decided to adopt an open approach to remove mineralization embedded within the proximal edge of a thickened PAL. Desmotomy of the PAL was performed in 15 horses (83.3%), including both horses in which the surgeon converted to an open approach following tenoscopic exploration.

### 3.1. Lesion Distribution

**Figure 3:**
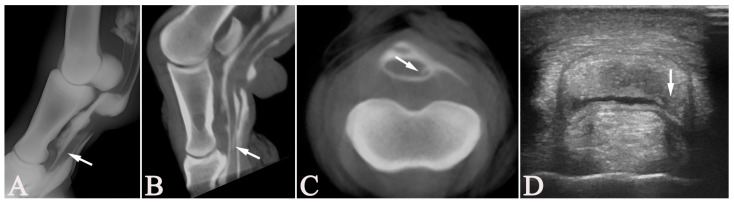
A 3-year-old Franches-Montagnes mare (Case 12) with a longitudinal tear (arrows) in the deep digital flexor tendon on the left hind pastern region, unobserved on tenoscopy. The lateromedial tenograph (**A**); the parasagittal (**B**) and transverse (**C**) multiplanar-reconstructed cone-beam computed tomographic tenographic (CTT) images; and the transverse ultrasonographic image (**D**) show this tear, located in the medial border of the tendon at the distal aspect of the proximal phalanx. (**A**,**B**) Dorsal = left, proximal = up; (**C**,**D**) lateral = left, plantar = up.

The following lesions were diagnosed on all modalities combined: RXT, US, CTT, and tenoscopy. A total of 15 DDFT lesions were diagnosed in 13 horses, with 12 (80%) involving the lateral aspect of the tendon and three (20%) involving the medial aspect of the tendon. In the metacarpal/-tarsal region, all but one DDFT lesion involved the lateral aspect of the tendon in all modalities. Two of these medial lesions were observed in the medial lobe of the DDFT in the pastern region on US and CTT (Figure 3), in addition to a lateral tear in the metatarsal region. More DDFT lesions (8/13; 61.5%) were present in the forelimbs than in the hindlimbs (5/13; 38.5%). Likewise, of the 12 horses diagnosed with SDFT lesions, these were predominantly on the lateral aspect of the tendon (9/14; 64.9%) vs. medially in 4/14 (28.6%). Of these, two horses had both medial and lateral lesions observed within the SDFT; one horse was diagnosed with a plantar midline lesion only. The fore- to hindlimb distribution was even for SDFT lesions, with 6/12 (50%) present in forelimbs and 6/12 (50%) in hindlimbs. A total of 14 changes within the MF were present in 13 horses and more frequently on the lateral aspect (11/14; 78.6%). In one horse, a lesion of the MF was only diagnosed on RXT, and therefore, the side could not be determined. In another horse, a lateral lesion was diagnosed with CTT, but with US and tenoscopy, a medial lesion was diagnosed. Lesions of the MF were almost equally distributed between fore- and hindlimbs, with 7/13 (53.6%) present in forelimbs and 6/13 (46.2%) in hindlimbs.

Supplementary Table S2 provides a more detailed overview of the lateralization of the lesions in the different modalities.

### 3.2. Inter-Modality Agreement

Table 1 provides an overview of the lesion distribution for the different modalities.

CTT detected the highest number of lesions in the DDFT, SDFT, and MF compared to all other modalities, closely followed by tenoscopy and US. With four cases, PALco was most diagnosed during tenoscopy, closely followed by US in three cases.

**Figure 4:**
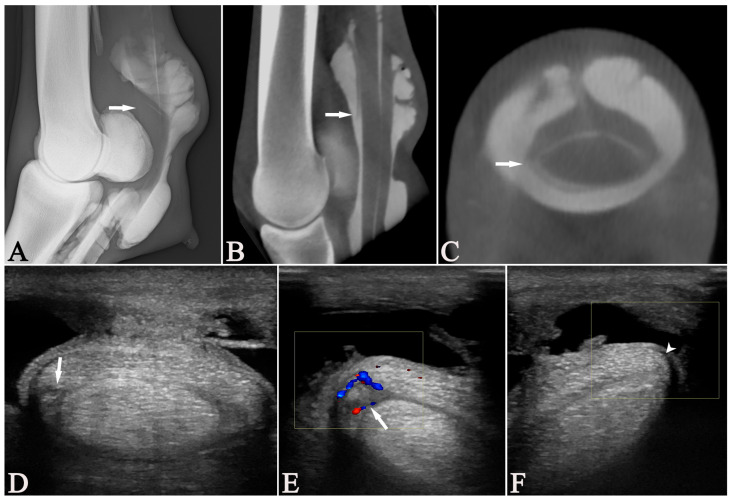
An 11-year-old Dutch Warmblood mare (Case 11) with a partial proximal manica flexoria (MF) tear (arrows) of the left forelimb, unobserved on the lateromedial tenograph (**A**). Two distally tapering contrast lines delineating the MF just proximal to proximal sesamoid bones appeared present at the dorsal border of DDFT on the lateromedial tenograph (**A**). However, the parasagittal (**B**) multiplanar-reconstructed cone-beam computed tomographic tenographic (CTT) image showed a mild dorsal displacement of the distal border of the MF. This displacement and an irregular lateral border of the MF were observed in the transverse multiplanar reconstructed CTT image (**C**) and seen as fibrillation on tenoscopy. On the transverse ultrasonographic image (**D**), a linear hypoechoic lesion was observed in the lateral aspect of the MF. This area showed an increased vascularization on color Doppler evaluation (**E**) that was not present in the medial aspect of the MF (arrowhead) (**F**). (**A**,**B**) Dorsal = left, proximal = up; (**C**,**D**) lateral = left, palmar = up.

**Figure 5:**
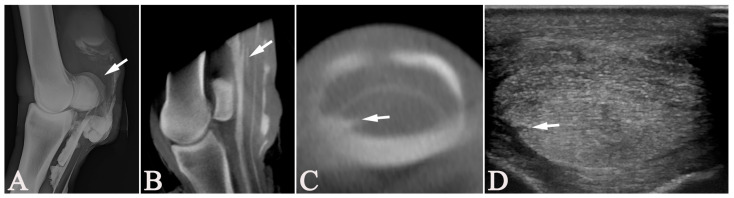
An 11-year-old Anglo-Arab gelding (Case 14) with a longitudinal tear (arrows) in the deep digital flexor tendon of the right forelimb, observed in all modalities. The lateromedial tenograph (**A**); the parasagittal (**B**) and transverse (**C**) multiplanar-reconstructed cone-beam computed tomographic tenographic (CTT) images; and the transverse ultrasonographic image (**D**) show this tear, located in the distal metacarpal region. The ultrasonography, CTT and tenoscopy agreed on its lateral localisation. (**A**,**B**) Dorsal = left, proximal = up; (**C**,**D**) lateral = left, palmar = up.

**Figure 6:**
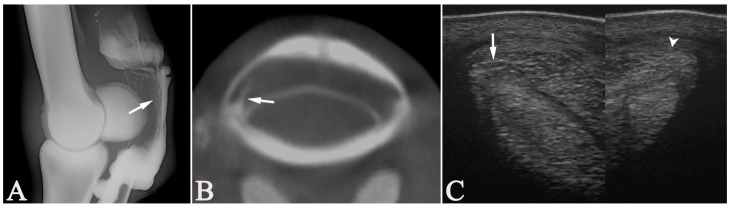
An 11-year-old Holstein gelding (Case 1) with a longitudinal tear (arrows) in the superficial digital flexor tendon (SDFT) of the right forelimb, observed in all modalities. The lateromedial tenograph (**A**); the transverse (**B**) multiplanar-reconstructed cone-beam computed tomographic tenographic (CTT) image; and the transverse ultrasonographic image (**C**) show this tear, located in the distal metacarpal region. The left side of the composed ultrasonographic image (**C**) shows the lesion as two parallel hyperechoic lines with the hypoechoic center in the lateral aspect of the SDFT using a palmarolateral approach. In comparison, the right image shows the normal medial aspect of the SDFT (arrowhead) using a palmaromedial approach. This lateral SDFT lesion was also diagnosed on tenoscopy. (**A**) Dorsal = left, proximal = up; (**B**,**C**) lateral = left, palmar = up.

**Figure 7:**
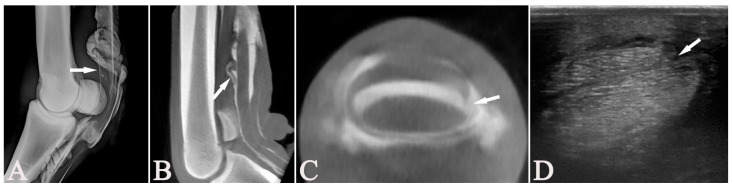
A 15-year-old Swiss Warmblood gelding (Case 17) with a proximal manica flexoria (MF) tear (arrows) of the left hindlimb, observed in all modalities. While there is a lack of double contrast contour dorsal to the deep digital flexor tendon on the lateromedial tenograph (**A**), typical for complete MF tears, the MF is visible as a folded structure on the parasagittal (**B**) multiplanar-reconstructed cone-beam computed tomographic tenographic (CTT) image. The transverse (**C**) CTT image, as well as the transverse ultrasonographic image (**D**) and the tenoscopic examination, showed a complete medial tear of the MF. (**A**,**B**) Dorsal = left, proximal = up; (**C**,**D**) lateral = left, plantar = up.

In only one case, with an MF lesion, all the modalities (US, RXT, CTT, and tenoscopy) agreed on the presence or absence of lesions in all structures. When RXT was excluded, the other modalities agreed in two additional cases (3/18), one where lesions were found within the DDFT, SDFT, and MF (Figure 4), and one with only an SDFT lesion. Regarding the presence or absence of lesions involving structures within the DFTS (DDFT, SDFT, MF), all modalities agreed in two cases, one of which was the MF lesion, and the other was a case in which US and tenoscopy had diagnosed a PALco but no other lesions. When RXT was excluded, the remaining modalities agreed in three additional cases (5/18) for findings involving structures within the DFTS. Specifically, in two cases, lesions in the DDFT, SDFT, and MF were diagnosed, and in one, only the SDFT was abnormal on US, CTT, and tenoscopy. For the evaluation of the DDFT, all modalities agreed in seven cases (Figure 5), for the SDFT in nine cases (Figure 6), and for the MF in nine cases (Figure 7), but in none of the cases for PALco. When RXT was excluded, the assessment matched in all modalities regarding the DDFT in 13 cases, the SDFT in 12 cases, and the MF in 13 cases, but still none regarding the presence of PALco.

The three horses (horses 1, 3 and 12) with a longer interval between the diagnostic imaging and the tenoscopic examination had seven lesions affecting the DDFT, SDFT and/or MF observed with CTT, with only a single DDFT lesion in one horse not observed on tenoscopy.

Agreement was calculated between all modalities for the lesion localization in the SDFT, DDFT, and MF, as well as for PALco. Details on the matching proportions and the agreement statistics (McNemar and Cohen’s Kappa) between the different imaging modalities for each defined lesion localization are presented in Table 2.

#### 3.2.1. Deep Digital Flexor Tendon

A significant difference was found between the diagnosis of DDFT lesions with RXT compared to US, CTT, and tenoscopy. Regarding the assessment of the DDFT, CTT agreed with US, US+RXT combined, and tenoscopy in 83.3% cases. These showed the highest κ of 0.65, which indicates a good agreement and was also the highest agreement for this structure. In all other comparisons, diagnoses matched between 66.7% and 44.4%. Matching diagnoses of 66.7% were observed for US alone or in combination with RXT and tenoscopy. The comparison between RXT and CTT showed 50% matching diagnoses.

#### 3.2.2. Superficial Digital Flexor Tendon

**Figure 8:**
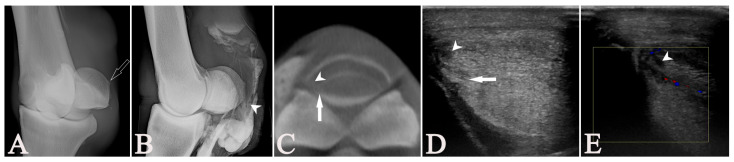
The 11-year-old Anglo-Arab gelding (Case 14) shown in Figure 5 had an additional, smaller tear (arrowheads) in the superficial digital flexor tendon (SDFT) of the right forelimb that was unobserved during the original ultrasonographic evaluation. A well-defined enthesophyte (open arrow) is present on the lateral proximal sesamoid bone at the insertion of the palmar annular ligament on the DL-PaMO projection of the fetlock (**A**), also seen on the lateromedial tenograph (**B**). At this level and extending distally, a lesion is observed within the SDFT on both the radiographic (**B**) and multiplanar-reconstructed cone-beam computed tomographic tenographic (CTT) images (**C**). Upon retrospective review of a video made during the ultrasonographic examination (US) for preparation of this paper, a small hypoechoic lesion (arrowheads) was observed in B-mode (**D**) and on color Doppler ultrasonography (**E**). Note the presence of the immediately adjacent DDFT lesion (arrows) visible on CTT and US, likely distracting the focus during the US examination and the reason why this lesion was unobserved originally. (**A**,**B**) Dorsal = left, proximal = up; (**C**,**D**) lateral = left, palmar = up.

Regarding the assessment of the SDFT, a significant difference was found comparing RXT with CTT or with tenoscopy. CTT and tenoscopy showed an excellent agreement for this structure, reaching the highest κ value (κ: 0.89) and 94.4% matching diagnoses (Figure 8). Tenoscopy compared to US or RXT showed 72.2% matching diagnoses in both and a κ of 0.44, which indicates moderate agreement. The comparisons of RXT with CTT and US with CTT or RXT all attained 66.7% matching for SDFT diagnoses, but κ values between 0.37 and 0.29, which indicate a poor agreement. The results of US+RXT compared to CTT and tenoscopy indicated that diagnoses matched in 72.2% (κ: 0.44) and 77.8% (κ: 0.56) of cases, respectively. These indicate moderate agreement.

#### 3.2.3. Manica Flexoria

Regarding the assessment of the MF, comparing CTT and tenoscopy attained the highest κ value (0.67), indicating a good agreement and 83.3% matching diagnoses. The comparison of CTT with RXT showed a slightly lower percentage of 77.8% matching diagnoses and moderate agreement (κ: 0.56). Moderate agreement (κ: 0.44) was also found when comparing US with CTT and RXT with tenoscopy, both of which had 72.2% of results matching. When comparing US to tenoscopy or RXT, diagnoses matched in 66.7% and 61.1%, respectively, but this was found to be a poor agreement with κ values of 0.33 and 0.22. The results of US+RXT, in comparison to CTT, yielded 77.8% matching diagnoses and moderate agreement (κ: 0.44). When US+RXT was compared with tenoscopy, good agreement with a κ of 0.67 was observed, and 83.3% of the diagnoses matched.

#### 3.2.4. Palmar/Plantar Annular Ligament Constriction

Cohen’s Kappa coefficient could not be calculated for comparisons between CTT and other modalities, as no cases of PALco were identified on CTT. The absence of variation in CTT results precluded statistical agreement analysis, and therefore, a meaningful statistical analysis was not possible. When combining all modalities, PALco was observed in only five horses. A significant difference (*p*-value of 0.046) was found when comparing the results of CTT with tenoscopy or with US+RXT, both of which had 77.8% of diagnoses matching. CTT and RXT had 94.4% matching diagnoses, but these all were negative. US+RXT and tenoscopy showed a good agreement (κ: 0.68), with 88.9% of the PALco diagnoses matching, including three positive cases. US alone, compared to tenoscopy, showed moderate agreement (κ: 0.47) with 83.3% of matching diagnoses, including two positive cases. Similarly, comparing US and CTT reached a value of 83.3%; however, these were all negative. Comparing RXT with tenoscopy or US, poor agreement was observed in both (κ values 0.34 and −0.09), with 83.3% and 77.8% matching results and 1 or 0 positive cases matching, respectively.

## 4. Discussion

This study compares and quantifies the agreement of US, RXT, standing CTT, and tenoscopy for the diagnosis of naturally occurring DDFT, SDFT, and MF pathologies within the DFTS and PALco. In the present study, more lesions in the DDFT, SDFT, and MF were diagnosed by means of CTT than by US, RXT, or tenoscopy. However, CTT was not reliable in diagnosing PALco. For each analyzed anatomical structure within the DFTS, the inter-modality agreement between CTT and tenoscopy showed the highest results. This supports the hypothesis that CTT diagnoses the most lesions within the DFTS and has the highest agreement with both tenoscopy and US, but it is not useful for diagnosing PALco.

Although previous DFTS studies mostly focused on the determination of Se and Sp for imaging modalities, the use of tenoscopy as a gold standard for the diagnosis of flexor tendon lesions has never been established. As mentioned by Nelson and colleagues, the results of such a comparison study should not be over-interpreted by inferring superiority of one diagnostic method over another but should demonstrate a general comparison between the diagnostic methods for lameness localized to the DFTS [35]. Therefore, all comparisons were made between all diagnostic methods used in this study.

### 4.1. Deep Digital Flexor Tendon

Although CTT achieved good agreement with both tenoscopy and US (83.3%), it also detected additional lesions not observed on US or tenoscopy. The three lesions unobserved by US but seen on CTT and tenoscopy were lateral marginal or longitudinal tears. These tears were located just distal to the proximal sesamoid bones in two horses (Cases 4 and 5). The third lesion was in an Icelandic horse (Case 9), with none of its lesions observed ultrasonographically due to poor image quality.

In three cases, DDFT lesions unobserved during tenoscopy were seen on US and CTT. These included two medial longitudinal tears localized in the pastern region at the distal aspect of the proximal phalanx (Cases 7 and 12), as shown in Figure 3. Both these horses had an additional lateral lesion of the MF and the DDFT near the MF. These additional, rather subtle lesions in the distal pastern were overlooked on tenoscopy. Plausible explanations are the limited maneuverability in the distal portions of the DFTS or that the operating surgeons focused on the more severe lesions proximal to the DFTS. The third unobserved DDFT lesion on tenoscopy was a small medial border lesion near the MF level (Case 17), located at the injection site for the tenography, which may have gone unnoticed or been considered iatrogenic and, therefore, not reported.

These observations explain why a poor agreement with 66.7% matching outcomes was found when comparing US or US+RXT to tenoscopy. When applying tenoscopy as the gold standard, other investigators have reported a wide range of Se and Sp for US, with Se varying from 35.4 to 88% and Sp from 71 to 100% [2,4,5,27,36]. Regarding those studies, it remains unknown if lesions were missed on tenoscopy that were correctly diagnosed by US.

In the inter-modality agreement, all comparisons for RXT with other modalities showed significant differences and poor agreement, with 44.4–61.6% of the outcomes matching. In previous studies, the Se and Sp of RXT were determined using tenoscopy as a gold standard and showed an Se ranging from 54 to 72% and an Sp from 53 to 84% [3,4,8]. Kent and colleagues found that DDFT lesions were commonly missed in RXT. This occurred more often in cases with merely a faint contrast column outlining the tendons in the fetlock canal, which might be caused by an over-extended metacarpo-/metatarsophalangeal joint [8]. In addition, they suggested that the superimposition of the proximal sesamoid bones over the region where DDFT lesions were likely to occur could explain the lower Se and Sp of RXT when compared to those of the MF [8]. Superimposition, however, is not an issue in standing CTT due to its cross-sectional nature and because the affected limb is not bearing weight during image acquisition when using the setup described here. This is considered the most likely explanation for the better Se and Sp for the diagnosis of DDFT lesions in this modality. In our study, RXT alone showed poor agreement with all compared modalities. The combined results of US+RXT did not lead to a better agreement with CTT or tenoscopy than with US alone, still showing a good agreement for CTT and a poor agreement for tenoscopy.

Our results indicate that CTT might have a greater diagnostic yield for DDFT lesions within the DFTS compared to tenoscopy or US, as it allowed for the detection of lesions that went unobserved in both other modalities. This indicates that a preoperative CTT will detect the vast majority of DDFT lesions found in tenoscopy, offering detailed information about lesion location, lesion morphology and lateralization.

### 4.2. Superficial Digital Flexor Tendon

In nine cases, all modalities agreed on the absence or presence of SDFT lesions. The inter-modality agreement between CTT and tenoscopy for detecting SDFT lesions was excellent, with 94.4% of the diagnoses matching, the highest in this study. We found moderate agreement when comparing tenoscopy to US and RXT, with both 72.2% of the outcomes matching. Comparing US to CTT, only 67% of matching diagnoses were found. Five of these horses had lesions unobserved on US but seen on CTT, as well as during tenoscopy in four cases (Cases 2, 9, 10, 12, and 14). In these four, the lesions observed in CTT and tenoscopy were located at the level of the PSB or just distal to them, with additional, more severe lesions in the DDFT in three and an MF lesion in the other one. The fifth horse (Case 7) had a lesion at the distal aspect of the proximal phalanx, unobserved in both US and tenoscopy, which had an additional DDFT lesion at the same level as well as an MF lesion.

Cender and colleagues evaluated the ability of US to diagnose SDFT lesions, reporting an Se of 66% and an Sp of 94%, using tenoscopy as a gold standard, which is similar to our results [4]. However, when comparing the combined results of US+RXT to tenoscopy and CTT, an increased, now moderate agreement was found in both, with 77.8% and 72.2% matching results, respectively. This is because a lesion in the SDFT was observed on RXT in two of the horses, in which lesions at the level of the PSB were not observed on US. This suggests that the combination of US and RXT could improve the diagnosis of SDFT lesions when CTT is not available. However, one should realize that even combining those modalities, fewer lesions will still be observed compared to CTT or tenoscopy.

It is likely that the more complex anatomy of the SDFT in the fetlock region, with the palmar/plantar location of the mesotenon, the dorsopalmar/-plantar flattening to the level of and splitting of the SDFT distal to the fetlock, makes recognizing abnormal contrast distribution on an LM RXT more difficult. The curvature of the SDFT at the level of the PSB and the presence of the spur at the proximal aspect of the pastern make this region challenging for US evaluation. In addition, the “satisfaction of search”, as well as US being a dynamic technique, makes small border lesions easily overlooked or wrongly interpreted if changes in an immediately adjacent structure are present. In addition, patient characteristics, such as poor skin penetration of the US beam, will influence the quality of the US examination. When revisiting the imaging during the preparation of the manuscript, an SDFT lesion that was missed in the initial US was noted in Case 14 (Figure 5 and Figure 8). The lesion was not counted, given the prospective nature of the study, but this case demonstrates the value of good imaging documentation and re-evaluation even in US. In another case, a focal thickening of the SDFT was observed on US, but not in any other modality (Case 10). Arguably, this kind of lesion could easily be missed by RXT and even tenoscopy. Another reason for the moderate performance of US may be the learning curve and experience of the observers; in the latter cases, fewer lesions seemed to be missed when compared to other modalities. Thus, CTT can be a valuable diagnostic tool for preoperative screening of SDFT lesions within the DFTS, providing information on lesion lateralization and morphology. Therefore, it may also enable better estimation of prognosis and treatment planning than US or RXT. However, when CTT is not available, combining US+RXT may be beneficial for evaluating the SDFT within the DFTS.

### 4.3. Manica Flexoria

For the assessment of MF tears, good agreement was also reported when comparing CTT to tenoscopy findings, with 83.3% matching results. As tenoscopy is considered the most reliable modality to detect MF lesions [29], our findings indicate that CTT is highly reliable for the detection or exclusion of MF lesions.

For this structure, RXT showed higher agreement with CTT and tenoscopy than US, with moderate agreement of 77.8% and 72.2%, respectively. The combined US+RXT results did not increase this moderate agreement compared to RXT alone.

Several studies have looked closer at diagnostic reliability in detecting MF tears using US, providing a wide range of Se values ranging from 23% to 92% but high Sp values of 92% to 94% [2,4,9,10,22]. Recently, the benefits of performing a dynamic US exam were reported for the diagnosis of MF tears [12,22]. In our study, limbs were examined ultrasonographically, both static (in a weight-bearing and non-weight-bearing position) and dynamically in a non-weight-bearing position. However, compression of the proximal DFTS outpouchings, as described by Garcia et al., was not performed [12] and could increase the diagnostic value of US examinations for diagnosing MF tears in the future. In the current study, full or partial tears of the MF, as well as fibrillation of the MF, were considered lesions. Mild irregularities of the MF, which do not include a rupture, are easily overlooked in most imaging modalities and are arguably best diagnosed by tenoscopy, as thickening of the synovial lining on tendons, as well as adjacent fibrillated fibers from other damaged structures, can hamper the diagnosis of these minor MF lesions with US and CTT.

Several studies using a similar protocol for the evaluation of RXT have reported Se values between 89% and 96% and Sp values ranging from 56% to 92%, taking tenoscopy as the gold standard [3,4,8]. This is similar to our observations.

Two recent studies used helical CT scanners to assess the diagnostic value of cross-sectional contrast studies of the DFTS in horses, one examining cadaveric limbs and the other horses under general anesthesia, but both attained high diagnostic values for helical CT [27,37]. In the case series by Shanklin and colleagues, CTT predicted the presence or absence of all tenoscopically confirmed lesions and their lateralization correctly [37]. Aßmann and colleagues reached an Se of 85% and an Sp of 96% for the diagnosis of previously artificially induced lesions in cadaveric limbs [27]. The lack of motion artifact in cadaveric limbs or horses under general anesthesia, and possibly the higher soft tissue resolution of a helical CT, may have improved the diagnostic yield compared to our study. In standing CTT, motion artifacts were previously speculated to be the most important contributor reducing the diagnostic yield [28], having the potential to confound pathological determination and needing diligent operators and compliant patients [38]. However, of the three cases where CTT and tenoscopy did not agree, one horse had a small partial tear of the MF, which was observed in US and tenoscopy but not in CTT. The other two cases had small partial tears diagnosed with CTT and US and one additionally in RXT, but no MF lesions were reported in tenoscopy. In none of these three cases was the MF lesion the only or main lesion observed. Specifically, in all modalities except RXT, most horses with MF lesions had additional lesions in the DDFT and/or SDFT, ranging from 77.8% to 90% of the horses. For these three modalities, 55.6% to 70% of the horses showed additional lesions in the DDFT and SDFT, and 11.1% and 22.2% were only in the SDFT and the DDFT, respectively. This is higher than previously reported, with Diekstall and colleagues finding 43% of horses with an MF tear having additional injuries to the SDFT [10] and Smith and Wright reporting that 23.7% of MF tears occur in combination with the DDFT and 8.7% with SDFT tears [2]. This difference possibly lies in a difference in reporting. As the subject of this study was the ability to detect lesions, all observed changes were reported, including minor fibrillation of a structure and not only those considered clinically significant.

For the standing CTT, hindlimb fetlocks were flexed during image acquisition, which led to a proximal displacement of the MF. Although this does not appear to have influenced the evaluation, an increased space between the SDFT and DDFT at the level of the MF was noticed in most hindlimbs. This may give the impression of an increased laxity or partial tear of the MF for inexperienced evaluators. On the other hand, an increased distance between the MF and the DDFT only occurred in horses where a tear in the MF was also observed on tenoscopy. This proximal displacement or increased space did not occur in weight-bearing RXT, nor was it reported in previous studies.

### 4.4. Palmar/Plantar Annular Ligament Constriction

For the PALco, Cohen’s Kappa coefficient could not be calculated for comparisons between CTT and the other modalities, as no cases of PALco were identified on CTT. Additionally, the numbers for PALco diagnoses were low, with four being reported during surgery, three in US, and one in RXT. Moderate agreement was reported when comparing US and tenoscopy, but the latter only diagnosed one additional case, with the same three that were observed on US. When the results of US and RXT were combined and compared to tenoscopy, good agreement was reached. Ultrasonography may provide a better impression as a dynamic examination, using flexion and extension of the limb in a non-weight-bearing position, can be performed [16,39]. A study comparing it to tenoscopy as a gold standard reported an Se of 68.9% and an Sp of 50% [36]. Even though the matching proportions of RXT and CTT were 94.4%, this could also indicate that RXT and CTT are equally unsuitable for the diagnosis of PALco, possibly due to their static nature. According to the results of Kent and colleagues, RXT lacks Se and Sp to diagnose PALco [8]. In a recent study, PALco was diagnosed by applying similar criteria to that of our study, using an atypical distribution of contrast throughout the sheath, a lack of contrast between the SDFT and PAL or a subjective impression of a flexor tendon constriction and/or a prominent flattening of the palmar or plantar surface of the SDFT at the level of the fetlock canal [38]. As the PAL is located outside the DFTS, the contrast cannot delineate its contours, and the poor contrast resolution of the CBCT compared to helical CT also did not allow for the evaluation of PAL thickening. However, no consensus has yet been described on how to reliably diagnose PALco from CTT images. Likewise, tenoscopy has its limitations in reliably diagnosing PALco, as it is heavily influenced by conformation of the distal extremity, particularly the length of the pastern and size of the heel bulbs, the horse breed, and by the position of the tenoscopic portal [40,41]. These influence the ease with which the rigid endoscope passes through the fetlock canal, which is subjectively assessed by the operating surgeon to determine the presence of PALco. Therefore, we conclude that further studies are needed to determine specific imaging findings that allow for the reliable diagnosis of PALco using CTT in a non-weight-bearing limb position. Clearly, the current results are unlikely to reflect the true absence or presence of PALco.

### 4.5. Modalities and Tenography Technique

Undoubtedly, scanning standing horses using a CBCT rather than a helical CT and imaging of the fetlock region in a non-weight-bearing position made image acquisition prone to motion artifacts. Although the latter hampered image quality in several CTT studies and cases, repeating the CBCT imaging until image volumes of diagnostic quality were generated resolved this problem in nearly all cases. In two cases, mild motion remained: in one, the image interpretation was not hampered, but in the other, the motion artifact led to a presumably erroneous diagnosis of an SDFT lesion, which was not found in any other modality. The participating board-certified radiologists also concurred that the cone-beam effect [40], inherent to CBCT imaging, did not negatively impact image interpretation. Furthermore, we speculate that having the examined limb in a non-weight-bearing position allowed for better contrast distribution, extending into lesions that reached the intrathecal surfaces. Compared to US, CTT image quality was also less hampered by patient factors, such as skin thickness, which can be notoriously increased in the pastern and fetlock of certain breeds, like cob-types and draft horses [37,42]. In Case 9, an Icelandic horse, mild to moderate thickening of the skin strongly impeded the US examination. In this horse, lesions in the DDFT, SDFT, and MF were not seen on US but diagnosed with CTT and tenoscopy. In two other cases (Cases 2 and 4), skin thickening was reported but was not considered a limiting factor, yet in Case 2, an SDFT was missed in US, and in Case 4, a DDFT lesion was missed in US, but these were diagnosed in CTT and tenoscopy. This suggests that the thickened skin may have limited the US assessment evaluation after all.

Compared to RXT, CTT allowed for the lateralization of the lesions and a better estimation of the extent of damage to each structure. Therefore, it would allow for optimal surgical planning, specifically regarding the recumbency of the horse. The value of performing a preoperative CT under general anesthesia has been shown to provide clinically valuable information in a short case series including four horses [37]. Ideally, this information becomes available before the horse is placed under general anesthesia, avoiding the need to reposition the horse prior to tenoscopy and an unnecessary prolongation of the anesthesia. Our observations match those of a recent study where CTT was thought to have the potential to combine the advantages of RXT with improved topography and lesion characterization of cross-sectional modalities and US, especially in thick-skinned breeds where US is often limited [38]. Yet US does have the benefit of providing a detailed image of the fibrillar pattern and core structure of tendons, which no other modality analyzed here can provide. The technique of performing a US examination is more user-dependent than the acquisition and interpretation of an RXT or CTT and, therefore, could hold an increased risk of false diagnoses. The application of Doppler US gives the benefit of seeing increased vascularization. It should ideally be used when performing a US examination of the non-weight-bearing limb, specifically in areas where tendon contours are unclear or a lesion diagnosis is uncertain. To improve the diagnostic yield of US, the authors recommend saving videos and images during the examination to allow for a retrospective analysis, possibly in combination with the later obtained diagnostic imaging. This allowed for the retrospective diagnosis of an SDFT lesion that had gone undetected during the initial examination and evaluation for the study (Figure 8). Since, under field conditions, CTT, unlike US and RXT, is usually not accessible, we added analyses combining the results of US and RXT to determine a possible improvement of agreement. However, these combined results only improved the agreement with CTT and tenoscopy for lesions of the SDFT. The combination of those modalities might, therefore, help with the diagnosis of SDFT lesions under field practice settings. For DDFT and MF lesions, the combination of US and RXT did not lead to an improved agreement compared to only US or RXT. To achieve a more complete evaluation of the structures contained within the DFTS with these modalities, both techniques still need to be applied. However, when the combined RXT and US observations are compared to CTT or tenoscopy for all structures within the DFTS combined (DDFT, SDFT and MF), matching diagnoses are only found in 44.4% and 38.9%, respectively, while 72.2% of matching diagnoses are found when CTT is compared to tenoscopy. It needs to be considered that higher agreement between modalities, no matter the localization, does not automatically mean the modalities are “better”; they may also miss or mis-detect the same lesions and, therefore, agree.

Despite being used for more than a decade, no consensus has yet been found on the ideal volume of contrast medium to be injected into the DFTS to optimize tenography and ensure the visualization of the MF [25]. The use of volumes greater than 35 mL may lead to leakage around the puncture site and cause discomfort to the horse [43]. Other investigators have reported that volumes of 15–17 mL reliably achieve sufficient distention to detect lesions [8], but it was speculated that the use of greater volumes might yield higher Se values on CTT [27]. Aßmann and colleagues found that a volume of 30 mL produced adequate DFTS distention and satisfying CTT studies in cadaveric specimens, whereas larger volumes only produced more leakage but did not improve image quality [27]. In the current study, a volume of 40 mL was used routinely. While this was well tolerated, it led to mild to moderate leakage in three cases, consequently hampering the ability to interpret RXT in one of these three cases. In two cases, a rapid drainage of contrast medium in the lymphatic vessels was observed and should not be misinterpreted as a possible lesion.

### 4.6. Limitations

The main limitation of this study is the small sample size of 18 horses, an issue often faced in clinical studies. This is highlighted by the wide CIs, which are linked to the small sample size, possibly leading to an underestimation of the agreement between modalities.

Moreover, neither radiologists nor surgeons were blinded to the previously acquired clinical or imaging findings for each case, which may have led to observer bias. This study aimed to mirror a standard diagnostic workflow rather than a blinded research setting. In such clinical settings, the radiologist and operating surgeon typically have access to the patient’s history and the results of prior imaging modalities. In addition, having several observers involved in a study is closer to real life, but the experience and performance of observers may vary and, therefore, create bias. No interobserver agreement or reliability assessment was performed for this study. Future studies with larger case numbers and/or assessing interobserver agreement would provide valuable additional information.

Another limitation of the present study is that video loops were not systematically recorded for either the ultrasonographic examinations or the tenoscopy, and in several cases, only still images of documented lesions or short videos of a few examined structures were available for review. Consequently, blinded re-evaluation of all ultrasonographic and tenoscopic examinations was not possible. Storage of video loops of all examined structures during ultrasonographic and tenoscopic examinations should be considered standard in the setup of future prospective study designs, so they are available for later re-evaluation.

A longer interval between imaging and tenoscopy was present in three horses, which could influence the agreement between modalities. However, in these horses, six of the seven lesions identified on CTT corresponded to the same anatomical structures affected during surgery. The single unobserved lesion on tenoscopy was a DDFT lesion in the pastern region of one horse. Therefore, the delay in the surgery is not considered to have influenced the agreement results in this study.

Tenoscopic findings were deliberately not set as the gold standard for the statistical analysis of the comparison with all other modalities. While tenoscopy is reliable for diagnosing superficial longitudinal tears of the flexor tendons [7], tears of the MF, and other lesions reaching the intrathecal surfaces within the DFTS [4], core lesions and other parenchymal lesions are not reliably visualized [31,36]. In our study, lesions of the DDFT at the level of the pastern were not observed in two cases during surgery since the affected area was not accessible with the standard portals (Figure 3). By principle, contrast-medium-based imaging, such as RXT and, to a lesser degree, CTT, is not more or less reliable in detecting lesions that do not reach the intrathecal surfaces of the structures contained within the DFTS, yet CTT can more easily detect volume changes, which may suggest intraparenchymal lesions due to its cross-sectional nature. Although tendinous core lesions at the level of the DFTS are rare in horses, US would be the modality of choice to diagnose such lesions. However, none of the horses included in the present study showed a core lesion on US. Therefore, no conclusion could be drawn concerning the reliability of diagnosing such lesions with CTT or any of the other modalities applied. In chronic cases, extensive adhesions may form [44], limiting tenoscopic exploration and leading to a reduced diagnostic yield.

Conversely, including all observed lesions as positive findings for that structure in every horse, independent of the number of modalities in which a lesion was observed, may have led to some overdiagnoses compared to the real in situ status of this structure in the horse. However, as lesions were reported in the same structure in multiple modalities for the vast majority of the reported changes, the number of overdiagnoses is likely small.

Finally, although magnetic resonance imaging often serves as the gold standard for the diagnosis of tendon lesions, it was not included in this study protocol. Hence, performing a similar study adding magnetic resonance imaging findings would be interesting, in addition to a comparison of high- and low-field magnetic resonance imaging, specifically for MF lesions.

## 5. Conclusions

The inter-modality agreement between CTT and tenoscopy reached the highest Cohen’s kappa and matching diagnoses for the DDFT, SDFT, and MF within the DFTS. For the examination of the DDFT, the agreement between US and CTT, and for the MF, the agreement between RXT and CTT, were high. These results indicate that CTT is the modality of choice for preoperative lesion detection within the DFTS in standing, sedated horses, combining some benefits of US and RXT. In combination with conventional imaging modalities like US and RXT, CTT is a useful addition to enhance preoperative screening and surgical planning.

## Figures and Tables

**Table 1:** Total counts of lesions within the DFTS detected with ultrasonography (US), positive contrast radiographic tenography (RXT), positive contrast computed tomographic tenography (CTT), and tenoscopy in the 18 horses with distention of the digital flexor tendon sheath. Abbreviations: DDFT, deep digital flexor tendon; SDFT, superficial flexor tendon; MF, manica flexoria; PAL, palmar/plantar annular ligament.

	Lesion	DDFT	SDFT	MF	PALco
Modality					
US		10	8	9	3
RXT		3	4	8	1
CTT		13	10	10	0
Tenoscopy		10	9	9	4

**Table 2:** Agreement statistics (McNemar and Cohen’s Kappa) between different imaging modalities for each lesion localization.

Lesion	Compared Modalities	*p*-Value	Proportion Matching	Proportion Non-Matching	Cohen’s Kappa	Agreement Kappa	Asymptotic SE	CI 95%
DDFT	US vs. RXT	0.01	61.1%	38.9%	0.28	poor	0.15	−0.01–0.57
	US vs. CTT	0.08	83.3%	16.7%	0.65	good	0.17	0.31–0.99
	US vs. Tsc	1.00	66.7%	33.3%	0.33	poor	0.22	−0.11–0.76
	RXT vs. CTT	0.001	44.4%	55.6%	0.14	poor	0.09	−0.04–0.33
	RXT vs. Tsc	0.02	50.0%	50.0%	0.07	poor	0.16	−0.24–0.38
	CTT vs. Tsc	0.08	83.3%	16.7%	0.65	good	0.17	0.31–0.99
	US+RXT vs. CTT	0.08	83.3%	16.7%	0.65	good	0.17	0.31–0.99
	US+RXT vs. Tsc	1.00	66.7%	33.3%	0.33	poor	0.22	−0.11–0.76
SDFT	US vs. RXT	0.10	66.7%	33.3%	0.29	poor	0.21	−0.11–0.69
	US vs. CTT	0.41	66.7%	33.3%	0.34	poor	0.21	−0.08–0.76
	US vs. Tsc	0.65	72.2%	27.8%	0.44	moderate	0.21	0.03–0.86
	RXT vs. CTT	0.01	66.7%	33.3%	0.37	poor	0.16	0.05–0.69
	RXT vs. Tsc	0.03	72.2%	27.8%	0.44	moderate	0.18	0.10–0.79
	CTT vs. Tsc	0.32	94.4%	5.6%	0.89	excellent	0.11	0.68–1.00
	US+RXT vs. CTT	0.65	72.2%	27.8%	0.44	moderate	0.21	0.03–0.86
	US+RXT vs. Tsc	1.00	77.8%	22.2%	0.56	moderate	0.20	0.17–0.94
MF	US vs. RXT	0.71	61.1%	38.9%	0.22	poor	0.23	−0.23–0.67
	US vs. CTT	0.65	72.2%	27.8%	0.44	moderate	0.21	0.03–0.86
	US vs. Tsc	1.00	66.7%	33.3%	0.33	poor	0.22	−0.10–0.77
	RXT vs. CTT	0.32	77.8%	22.2%	0.56	moderate	0.19	0.19–0.93
	RXT vs. Tsc	0.65	72.2%	27.8%	0.44	moderate	0.21	0.03–0.86
	CTT vs. Tsc	0.56	83.3%	16.7%	0.67	good	0.18	0.32–1.00
	US+RXT vs. CTT	0.32	77.8%	22.2%	0.54	moderate	0.20	0.15–0.93
	US+RXT vs. Tsc	0.18	72.2%	27.8%	0.44	moderate	0.20	0.05–0.84
PALco	US vs. RXT	0.32	77.8%	22.2%	−0.09	poor	0.07	−0.23–0.05
	US vs. CTT	0.08	83.3%	16.7%	N/A	N/A	N/A	N/A
	US vs. Tsc	0.56	83.3%	16.7%	0.47	moderate	0.26	−0.04–0.98
	RXT vs. CTT	0.32	94.4%	5.6%	N/A	N/A	N/A	N/A
	RXT vs. Tsc	0.08	83.3%	16.7%	0.34	poor	0.26	−0.02–0.85
	CTT vs. Tsc	0.046	77.8%	22.2%	N/A	N/A	N/A	N/A
	US+RXT vs. CTT	0.046	77.8%	22.2%	N/A	N/A	N/A	N/A
	US+RXT vs. Tsc	1.00	88.9%	11.1%	0.68	good	0.21	0.27–1.00

Each row shows the comparison between two imaging modalities used to evaluate the structure mentioned in the “Lesion” column. Reported *p*-values are from the results of McNemar’s test. The proportion matching/non-matching indicates where the compared modalities agreed (combined true positive and true negatives) or disagreed, respectively. Cells with N/A indicate that the comparison between modalities could not be calculated due to 0 counts in those combinations. Abbreviations: US, ultrasonography; RXT, positive contrast radiographic tenography; CTT, positive contrast computed tomographic tenography; US+RXT, ultrasonography and positive contrast radiographic tenography combined results; Tsc, tenoscopy; DDFT, deep digital flexor tendon; SDFT, superficial digital flexor tendon; MF, proximal manica flexoria; PALco, palmar/plantar annular ligament constriction N/A; not applicable; SE, standard error; CI, confidence interval.

## Data Availability

The original contributions presented in this study are included in the article/Supplementary Material. Further inquiries can be directed to the corresponding authors.

## Supplementary Materials

The following supporting information can be downloaded at https://www.mdpi.com/article/10.3390/vetsci13030268/s1: Table S1: Other pathologies found in imaging and tenoscopy of the metacarpal/-tarsal and pastern region in the 18 horses with digital flexor tendon sheath distention. Table S2: Lesion localization within the DFTS in 18 horses, using ultrasonography (US), positive contrast radiographic tenography (RXT), positive computed tomographic tenography (CTT) and tenoscopy.

## Abbreviations

The following abbreviations are used in this manuscript:

MF	Manica flexoria
DFTS	Digital flexor tendon sheath
CTT	Positive contrast computed tomographic tenography
RXT	Positive contrast radiographic tenography
US	Ultrasonography
DDFT	Deep digital flexor tendon
SDFT	Superficial digital flexor tendon
PAL	Palmar/plantar annular ligament
PALco	Palmar/plantar annular ligament constriction
Se	Sensitivity
Sp	Specificity
LM	lateromedial
DPa/DPl	Dorsopalmar/-plantar
DL-PaMO	Dorsal 45° lateral–palmaromedial oblique
DL-PlMO	Dorsal 45° lateral–plantaromedial oblique
DM-PaLO	Dorsal 45° medial–palmarolateral oblique
DM-PlLO	Dorsal 45° medial–plantarolateral oblique
CBCT	Cone-beam computed tomography
SE	Standard error
CI	Confidence interval
κ	Kappa
