# Supplementary material for: Comparison of Ultrasonography, Contrast Radiographic Tenography, Cone-Beam Computed Tomographic Tenography, and Tenoscopy for Lesion Detection Within the Digital Flexor Tendon Sheath of Horses—A Prospective Clinical Trial
Source: Vet Sci. 2026 Mar 13;13(3):268. doi: 10.3390/vetsci13030268 (PMC13029922; doi:10.3390/vetsci13030268)
Supplement: Supplementary file 1 [file vetsci-13-00268-s001.zip › vetsci-4154814-supplementary.pdf]

Supplemenentary table S1: Additional pathology found in ultrasonography, positive contrast radiographic tenography (RXT), cone beam computed tomographic tenography (CTT) and tenoscopy, excluding those of the deep digital flexor tendon (DDFT) and superficial digital flexor tendon (SDFT), manica flexoria (MF) and palmar/plantar annular ligament (PAL) constriction and thickening. Abbreviations: DFTS, digital flexor tendon sheath; MC, metacarpus; MT, metatarsus; PSB, proximal sesamoid bones; MIM, interosseus medius muscle; PDAL, proximal digital annular ligament; P1, proximal phalanx; P2, middle phalanx; ISL, intersesamoidean ligament; AL, accessory ligament of the DDFT; OSL, oblique sesamoidean ligament.

Horse	Anatomical structure	Diagnosis ultrasonography	Diagnosis RXT	Diagnosis CTT	Diagnosis tenoscopy
**1**	DFTS	Distention, tenosynovitis	-	Adhesions with SDFT	-
	MC III	-	Fragment sagittal ridge, osteoarthritis fetlock joint	Osteophytes MC III	-
**2**	DFTS	-	Subcutaneous ganglion of DFTS	Subcutaneous ganglion of DFTS	-
	PAL	Fibrosis with cystic degeneration and soft tissue mineralization	Fibrosis with mineralization	Mineralization	Mineralization
**3**	DFTS	Distention, Tenosynovitis, mesotenon thickening	Soft tissue swelling	Mesotenon thickening	-
**4**	DFTS	Distention, synovial proliferation	Soft tissue swelling	Synovial proliferation	-
	PSB	Irregular surface lateral PSB palmar	New bone formation lateral PSB palmar	New bone formation both PSB palmar	-
	MC III	-	Irregular sagittal ridge	-	-
**5**	DFTS	Distention, synovial proliferation	-	Filling defect likely caused by synovial proliferation	-
	MIM	Tendinopathy both branches	-	-	-
**6**	DFTS	Distention, tenosynovitis	Soft tissue swelling	Distention, tenosynovitis	-
	MIM	Tendinopathy both branches, focal defect lateral insertion	-	-	-
	PAL	Lateral insertion desmopathy	Insertion enthesopathy	-	-
	PSB	-	Distal half of lateral PSB irregular	Focal defect at MI insertion, irregular lateral SB at PAL insertion	-
	ISL	ISL desmopathy	-	ISL desmopathy	-
**7**	DFTS	Distention, synovial proliferation	Soft tissue swelling, tenosynovitis	Distention, tenosynovitis	-

	MIM	Tendinopathy and enthesopathy of branches	-	-	-
	PSB	Irregular at PAL insertion, defect at MIM insertion	Both irregular at PAL insertion	Smooth new bone formation on both PSB	-
**8**	DFTS	-	Soft tissue swelling	Soft tissue swelling	
	PDAL	Desmitis	-	-	-
	PSB	Enthesopathy at PAL insertion	Enthesophyte lateral PSB at PAL insertion	Enthesophyte lateral PSB at PAL insertion	-
	P2	-	Dorsoproximal remodeling	Irregular dorsoproximal	-
	MT III	-	Distal remodeling collateral ligament insertion	Distal remodeling collateral ligament insertion	-
**9**	DFTS	Distention, tenosynovitis	Soft tissue swelling	Distention, tenosynovitis	Synovial proliferation
	MI	Insertion desmopathy medial branch	-	-	-
	PSB	Enthesopathy at PAL insertion	Lateral irregular at PAL insertion	Lateral irregular	-
**10**	DFTS	Distention, tenosynovitis	Soft tissue swelling	-	-
	PSB	Both irregular palmar	Both irregular palmar	Both irregular palmar	-
**11**	DFTS	Distention, tenosynovitis	Soft tissue swelling	Distention, synovial proliferation	Tenosynovitis, synovial proliferation
	MI	Tendonitis medial branch with avulsion fragments / mineralizations	Mineralizations in lateral MI branch	-	-
	P1	-	Palmar new bone formation	Palmar new bone formation	-
	PSB	Irregular at insertion of ligaments and lateral MIM branch	Enthesophytes at insertion of medial MIM branch and PAL lateral insertion and OSL	Enthesophytes at insertion of medial MIM branch and oblique ISL, osteopathy of medial PSB	-
**12**	DFTS	Distention, synovial thickening, tenosynovitis	Soft tissue swelling	Soft tissue swelling, thickened dorsal synovial fold medially	Tenosynovitis, synovial proliferation
	PSB	Enthesophytes at PAL insertion	Enthesophytes at PAL insertion	Enthesophytes at PAL insertion	-
	PDAL	Desmitis	-	-	-
**13**	DFTS	Distention, tenosynovitis	Soft tissue swelling	-	-
	P2	-	Dorsoproximal new bone formation	Dorsoproximal new bone formation	-

**14**	DFTS	Distention, tenosynovitis, mineralization medial wall	Soft tissue swelling	Mineralization medial wall	-
	MI	Tendonitis medial branch	-	-	-
	AL	Tendonitis	-	-	-
	PSB	Enthesophytes at PAL insertion	Enthesophytes at PAL insertion	Enthesophytes at PAL insertion, remodeling	-
	P1	-	Enthesophytes at PDAL insertion, Osteophytes dorsomedial	Osteophytes dorsomedial	-
	P2	-	Osteophytes dorsoproximal	Osteophytes dorsomedial	-
	MC III	-	Mineralization of soft tissue	Mineralization of soft tissue	-
	PDAL	Desmitis	-	-	-
	OSL	-	-	Desmopathy lateral OSL	-
**15**	DFTS	Distention, tenosynovitis	Soft tissue swelling	Distention, tenosynovitis	-
	PAL	Desmopathy with median partial tear	-	-	-
	PSB	Enthesophytes at PAL insertion	Enthesophytes at PAL insertion	Enthesophytes at PAL insertion	Enthesophytes lateral PSB
**16**	DFTS	Distention	Soft tissue swelling	Distention, tenosynovitis	-
	MI	Tendinopathy both branches	-	-	-
	P1	-	Osteochondral fragment dorsal	Osteochondral fragment dorsal	-
**17**	DFTS	Distention, synovial proliferation	Soft tissue swelling	-	-
	MI	Tendinopathy both branches	-	-	-
	P1	-	Osteophytes plantarodistal	Osteophytes plantarodistal	-
	P2	-	Osteophytes dorsoproximal	Osteophytes dorsoproximal Subchondral cyst-like lesion	-
**18**	DFTS	Distention, synovial proliferation	-	-	-
	PSB	Enthesophytes at PAL insertion	Enthesophytes at PAL insertion	Enthesophytes at PAL insertion	-

Supplementary table S2: Lesion localization within the DFTS in 18 horses, using ultrasonography (US), positive contrast radiographic tenography (RXT), and positive computed tomographic tenography (CTT) in comparison to tenoscopy as the gold standard. All detected lesions were marginal lesions. Abbreviations: DDFT, deep digital flexor tendon; SDFT, superficial flexor tendon; MF, manica flexoria; PAL, palmar/plantar annular ligament; -, no lesion detected

**Horse**	**Structure**	**US**	**RXT**	**CTT**	**Tenoscopy**
**1**	DDFT	Lateral tear	Tear	Lateral tear	Lateral tear
	SDFT	Lateral & Midline tear	Tear	Lateral tear	Lateral tear
	MF	-	-	Lateral tear	Lateral tear
**2**	DDFT	-	-	-	-
	SDFT	-	-	Lateral tear	Lateral tear
	MF	-	-	-	-
**3**	DDFT	-	-	-	-
	SDFT	-	-	-	-
	MF	Lateral tear	Tear	Lateral tear	Lateral tear
**4**	DDFT	-	-	Lateral tear	Lateral tear
	SDFT	-	-	-	-
	MF	-	Tear	-	-
**5**	DDFT	-	-	Lateral tear	Lateral tear
	SDFT	-	-	-	-
	MF	-	-	-	-
**6**	DDFT	Lateral tear	-	Lateral tear	Lateral tear
	SDFT	Lateral tear	Tear	Lateral tear	Lateral tear
	MF	Lateral tear	Tear	Lateral tear	Lateral tear
**7**	DDFT	Mesotenon level: Lateral tear	-	Mesotenon level: Lateral tear	-
		Pastern level: Medial tear	-	Pastern level: Medial tear	-
	SDFT	-	-	Medial tear	-
	MF	Lateral tear	Tear	Lateral tear	-
**8**	DDFT	-	-	-	-
	SDFT	Midline tear	-	-	-
	MF	-	-	-	-
**9**	DDFT	-	-	Lateral tear	Lateral tear
	SDFT	-	-	Lateral tear	Lateral tear
	MF	-	Tear	Lateral tear	Lateral tear
**10**	DDFT	Lateral tear	-	Lateral tear	Lateral tear
	SDFT	-	-	-	-
	MF	Lateral tear	-	Lateral tear	-
**11**	DDFT	Lateral tear	-	Lateral tear	Lateral tear
	SDFT	Medial tear[1]	-	Lateral tear	Lateral tear
	MF	Lateral tear	-	Lateral tear	Lateral tear
**12**	DDFT	-	PAL region: Tear	PAL region: Lateral tear	-
		Pastern region: Medial tear	-	Pastern region: Medial tear	-
	SDFT	Medial & Midline tear	Tear	Lateral tear	Lateral tear
	MF	Medial tear	Tear	Lateral tear	Medial tear
**13**	DDFT	-	-	-	-
	SDFT	Medial tear	-	Medial tear	Medial tear
	MF	-	-	-	-
**14**	DDFT	Lateral tear	Tear	Lateral tear	Lateral tear

	SDFT	-	Tear	Lateral tear	Lateral tear
	MF	-	Tear	Lateral tear	Lateral tear
**15**	DDFT	Lateral tear	-	Lateral tear	Lateral tear
	SDFT	Lateral tear	-	Lateral tear	Lateral tear
	MF	Lateral tear	-	-	Lateral tear
**16**	DDFT	Lateral tear	-	Lateral tear	Lateral tear
	SDFT	-	-	-	-
	MF	Lateral tear	-	-	-
**17**	DDFT	Medial tear	-	Medial tear	-
	SDFT	-	-	-	-
	MF	Medial tear	Tear	Medial tear	Medial tear
**18**	DDFT	-	-	-	-
	SDFT	-	-	-	-
	MF	-	-	-	-

[1] both a medial and lateral tear observed on US
